# Gadofosveset trisodium in coronary magnetic resonance angiography at 3 Tesla

**DOI:** 10.1186/1532-429X-16-S1-P158

**Published:** 2014-01-16

**Authors:** Fabio S Raman, Mark A Ahlman, Jianing Pang, Filip Zemrak, Debiao Li, David Bluemke

**Affiliations:** 1 Department of Radiology and Imaging Sciences, National Institutes of Health, Bethesda, Maryland, USA; 2 Molecular Biomedical Imaging Laboratory, National Institute of Biomedical Imaging and Bioengineering, Bethesda, Maryland, USA; 3 Biomedical Imaging Research Institute, Cedars Sinai Medical Center, Los Angeles, California, USA; 4 Centre for Advanced Cardiovascular Imaging, William Harvey Research Institute, Queen Mary, University of London, Barts and The London National Institute for Health Research Biomedical Research Unit, London, UK; 5 Departments of Radiology and Biomedical Engineering, Northwestern University, Chicago, Illinois, USA

## Background

Coronary magnetic resonance angiography (MRA) at 3T suffers from imaging inconsistencies compared to 1.5T despite the use of gadolinium-based contrast agents (GBCAs). Gadofosveset Trisodium (Ablavar^®^, Lantheus Medical Imaging), with its high relaxivity and long intravascular residence time, offers greater potential over standard GBCAs to improve evaluation of the coronary arteries. Compared to its validation in larger arteries, the optimum dosage for coronary MRA has yet to be assessed. The purpose of the study was to evaluate the diagnostic potential of a 0.06 mmol/kg dose of Gadofosveset compared to a standard clinical dose of 0.03 mmol/kg, using a free-breathing whole-heart coronary MRA protocol with 1.0 mm3 spatial resolution and 100% navigator efficiency. The injection protocol was optimized for the prolonged pharmacokinetics of Gadofosveset.

## Methods

Twenty two contrast enhanced CMR scans were performed in 11 subjects [2 (18.2%) male; 27.3 ± 6 years; BMI = 23.1 ± 3 kg/m2] on a 3.0T Verio Siemens scanner, using an inversion-prepared spoiled gradient-echo sequence (modified from Ref. [1]). The two scans were separated by a 30-60 day interval, using dosages of either 0.06 mmol/kg or 0.03 mmol/kg of Gadofosveset. Quantitatively, signal-to-noise ratio (SNR) and contrast-to-noise ratios (CNR) were measured. Qualitative AHA quality scores were evaluated [2]. Pairwise, Student's t-test and Wilcoxon rank test were performed for quantitative and qualitative assessment (MedCalc Software v12.2.1, MariaKerke, Belgium).

## Results

**Figure 1:**
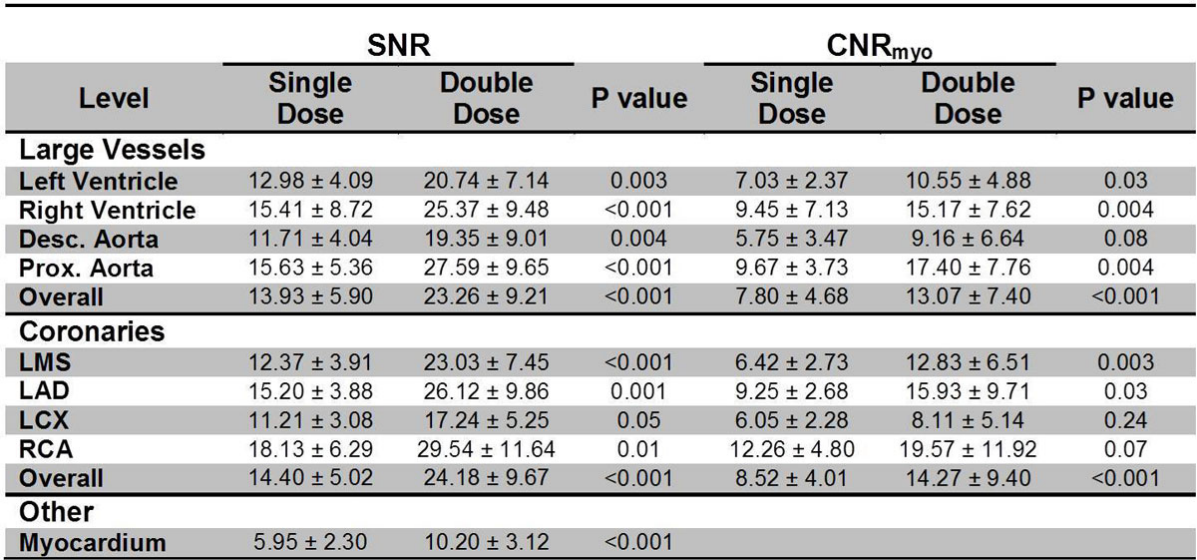
**Data is presented as mean ± SD**. CNRmyo: CNR between the blood and the myocardium. LMS: left main stem. LAD: left anterior descending coronary artery. LCX: left circumflex coronary artery. RCA: right coronary artery. Desc. Aorta: descending Aorta. Prox. Aorta: proximal aorta

**Figure 2:**
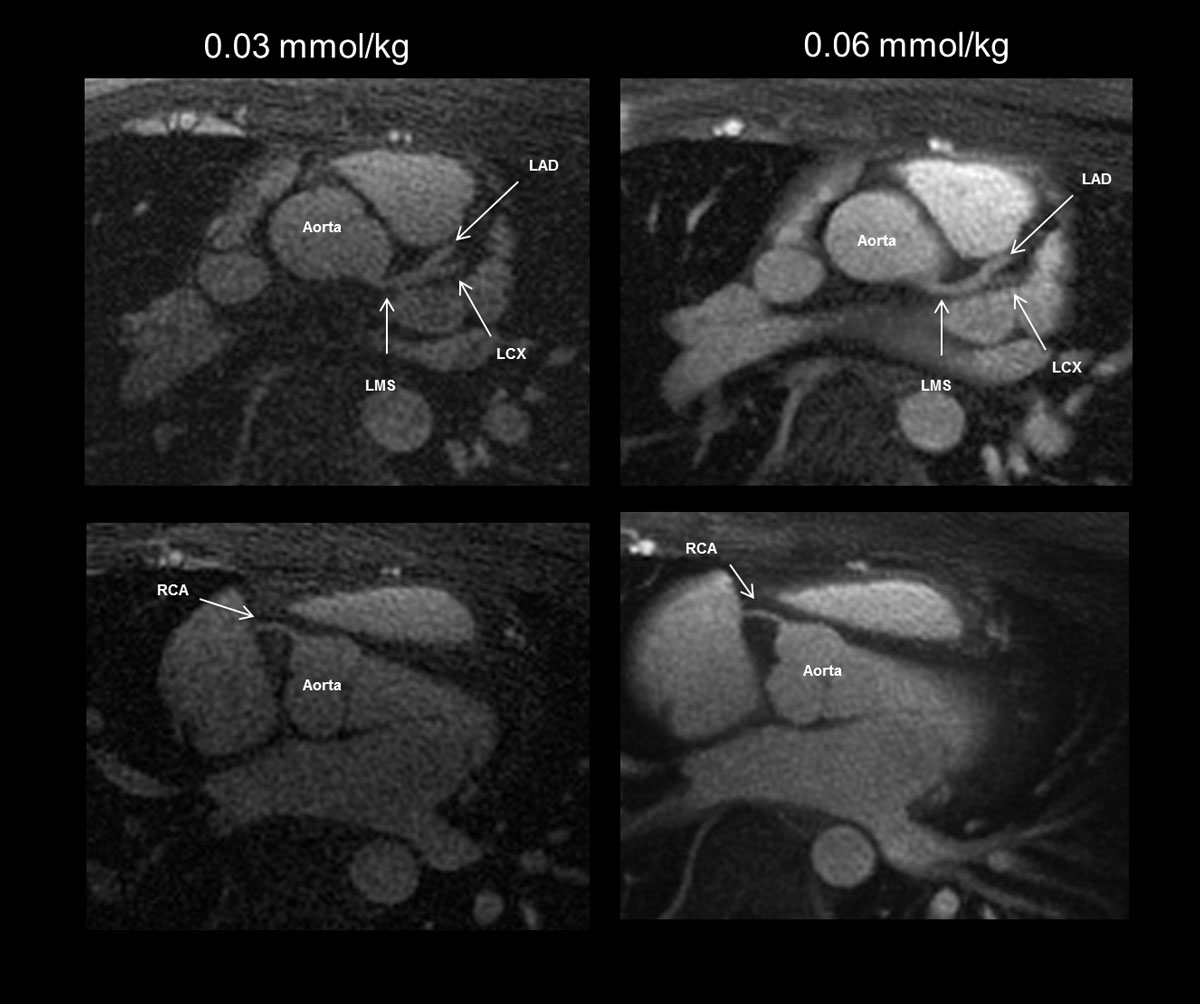
**Axial slices through the heart from a healthy study subject at a single- and double-dose of Gadofosveset**. Arrows indicate the locations of the ROI placement for measurement of the proximal sections of the coronary arteries. The aorta, left main stem (LMS), left anterior decending (LAD), left circumflex LCX,and right coronary artery (RCA) are shown.

Overall, SNR and CNR was higher (p < 0.001) in the coronary arteries for double- over single-dose of Gadofosveset (24.18 ± 9.67 vs. 14.40 ± 5.0 and 14.27 ± 9.40 vs. 8.52 ± 4.01, respectively, Figure 1). Individual coronary arteries demonstrated greater SNR enhancement for 0.06 mmol/kg vs. 0.03 mmol/kg for the LMS (23.03 ± 7.45 vs. 12.37 ± 3.91, p < 0.001), LAD (26.12 ± 9.86 vs. 15.20 ± 3.88, p = 0.001), and RCA (29.54 ± 11.64 vs. 18.13 ± 6.29, p = 0.01). CNR comparisons revealed similar results. Qualitatively, similar number of main and branch vessels were identified by two reviewers (Figure 2).

## Conclusions

Quantitatively, a double dose of Gadofosveset shows improvement in coronary arterial enhancement over clinical dose. Ongoing research is aimed at evaluating the diagnostic efficacy of a double-dose scanning regimen.

## Funding

Funded by the National Institutes of Health (NIH) Intramural program.
